# Hexaaqua­zinc(II) bis­(4-hydroxy­benzene­sulfonate) dihydrate

**DOI:** 10.1107/S1600536809048375

**Published:** 2009-11-21

**Authors:** Shan Gao, Seik Weng Ng

**Affiliations:** a College of Chemistry and Materials Science, Heilongjiang University, Harbin 150080, People’s Republic of China; b Department of Chemistry, University of Malaya, 50603 Kuala Lumpur, Malaysia

## Abstract

The asymmetric unit of the title hydrated mol­ecular salt, [Zn(H_2_O)_6_](C_6_H_5_O_4_S)_2_·2H_2_O, contains two half-cations, two anions and two uncoordinated water mol­ecules. Both cations are completed by crystallographic inversion symmetry, generating almost regular ZnO_6_ octa­hedra. In the crystal, the cations, anions and uncoordinated water mol­ecules are linked by O—H⋯O hydrogen bonds, forming a three-dimensional network.

## Related literature

For the isostructural cobalt and nickel analogs of the title compound, see: Du *et al.* (2007 ▶) and Kosnic *et al.* (1992 ▶), respectively.
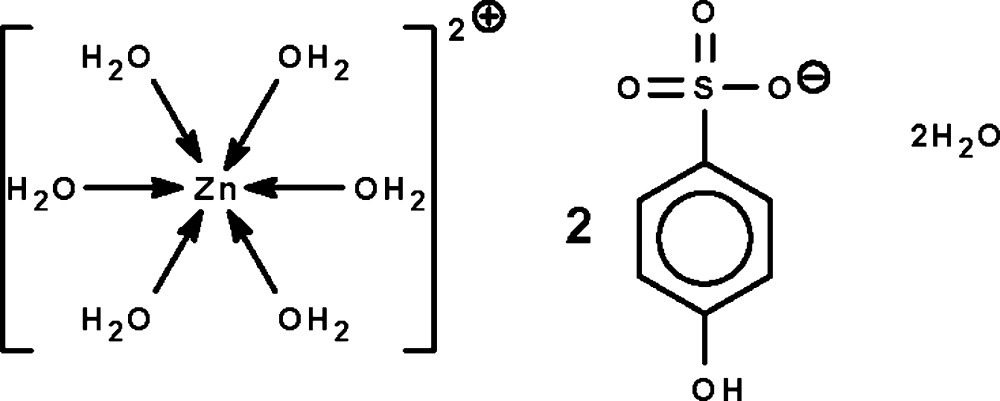


## Experimental

### 

#### Crystal data

[Zn(H_2_O)_6_](C_6_H_5_O_4_S)_2_·2H_2_O
*M* *_r_* = 555.82
Monoclinic, 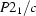

*a* = 11.7957 (5) Å
*b* = 7.2590 (4) Å
*c* = 25.3992 (11) Å
β = 94.340 (1)°
*V* = 2168.57 (18) Å^3^
*Z* = 4
Mo *K*α radiation
μ = 1.40 mm^−1^
*T* = 293 K
0.19 × 0.19 × 0.15 mm

#### Data collection

Rigaku R-AXIS RAPID IP diffractometer
Absorption correction: multi-scan (*ABSCOR*; Higashi, 1995 ▶) *T*_min_ = 0.777, *T*_max_ = 0.817
20669 measured reflections
4940 independent reflections
3319 reflections with *I* > 2σ(*I*)
*R*_int_ = 0.040

#### Refinement

*R*[*F* ^2^ > 2σ(*F* ^2^)] = 0.030
*wR*(*F* ^2^) = 0.082
*S* = 1.03
4940 reflections
355 parameters
18 restraints
H atoms treated by a mixture of independent and constrained refinement
Δρ_max_ = 0.33 e Å^−3^
Δρ_min_ = −0.40 e Å^−3^

### 

Data collection: *RAPID-AUTO* (Rigaku, 1998 ▶); cell refinement: *RAPID-AUTO*; data reduction: *CrystalClear* (Rigaku/MSC, 2002 ▶); program(s) used to solve structure: *SHELXS97* (Sheldrick, 2008 ▶); program(s) used to refine structure: *SHELXL97* (Sheldrick, 2008 ▶); molecular graphics: *X-SEED* (Barbour, 2001 ▶); software used to prepare material for publication: *publCIF* (Westrip, 2009 ▶).

## Supplementary Material

Crystal structure: contains datablocks global, I. DOI: 10.1107/S1600536809048375/hb5229sup1.cif

Structure factors: contains datablocks I. DOI: 10.1107/S1600536809048375/hb5229Isup2.hkl

Additional supplementary materials:  crystallographic information; 3D view; checkCIF report

## Figures and Tables

**Table 1:** Selected bond lengths (Å)

Zn1—O2*W*	2.0654 (16)
Zn1—O3*W*	2.0692 (16)
Zn1—O1*W*	2.1087 (15)
Zn2—O4*W*	2.0763 (15)
Zn2—O6*W*	2.0780 (16)
Zn2—O5*W*	2.0849 (16)

**Table 2:** Hydrogen-bond geometry (Å, °)

*D*—H⋯*A*	*D*—H	H⋯*A*	*D*⋯*A*	*D*—H⋯*A*
O1w—H11⋯O1	0.84 (1)	1.93 (1)	2.763 (2)	169 (3)
O1w—H12⋯O7w	0.85 (1)	1.86 (1)	2.707 (2)	175 (2)
O2w—H21⋯O2	0.85 (1)	1.95 (1)	2.792 (2)	172 (3)
O2w—H22⋯O4^i^	0.85 (1)	1.92 (1)	2.745 (2)	166 (3)
O3w—H31⋯O3^ii^	0.84 (1)	1.97 (1)	2.800 (2)	168 (3)
O3w—H32⋯O7w^ii^	0.84 (1)	1.95 (1)	2.790 (2)	174 (3)
O4w—H41⋯O5	0.84 (1)	1.91 (1)	2.742 (2)	174 (3)
O4w—H42⋯O1w^iii^	0.85 (1)	2.08 (1)	2.914 (2)	167 (3)
O5w—H51⋯O6	0.85 (1)	1.91 (1)	2.755 (2)	175 (3)
O5w—H52⋯O8^iv^	0.85 (1)	1.98 (1)	2.819 (2)	168 (3)
O6w—H61⋯O8w	0.85 (1)	1.85 (1)	2.698 (2)	170 (3)
O6w—H62⋯O7^v^	0.84 (1)	2.03 (1)	2.8401 (19)	163 (3)
O4—H4⋯O7^i^	0.85 (1)	1.93 (1)	2.777 (2)	175 (3)
O8—H8⋯O3^i^	0.85 (1)	1.95 (1)	2.788 (2)	172 (3)

Symmetry codes: (i) 
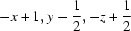
; (ii) 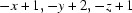; (iii) 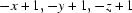; (iv) 
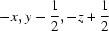
; (v) 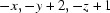.

**Fig. 1.**
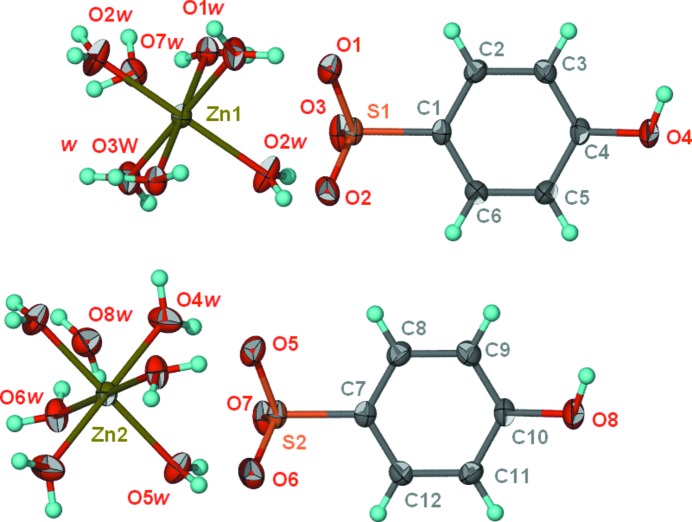
The molecular structure of (I) shown at the 50% probability level; hydrogen atoms are drawn as spheres of arbitrary radius. Unlabelled atoms bonded to Zn1 and Zn2 are generated by the symmetry operations (1–x, 1–y, 1–z) and (–x, 1–y, 1–z), respectively.

## supplementary crystallographic information

### Experimental

One millimolar quantities each of zinc dichloride and *p*-hydroxylbenzenesulfonic acid were dissolved in water; colourless prisms of (I) were isolated after a few days.

### Refinement

Carbon-bound H-atoms were placed in calculated positions (C—H = 0.93 Å) and were included in the refinement in the riding model approximation, with *U*_iso_(H) = 1.2*U*_eq_(C). The water H-atoms were located in a difference Fourier map, and were refined with a distance restraint of O–H = 0.85±0.01 Å; their U_iso_ values were refined.

### Crystal data

**Table:**

[Zn(H_2_O)_6_](C_6_H_5_O_4_S)_2_·2H_2_O	*F*(000) = 1152
*M**_r_* = 555.82	*D*_x_ = 1.702 Mg m^−^^3^
Monoclinic, *P*2_1_/*c*	Mo *K*α radiation, λ = 0.71073 Å
Hall symbol: -P 2ybc	Cell parameters from 12326 reflections
*a* = 11.7957 (5) Å	θ = 3.2–27.5°
*b* = 7.2590 (4) Å	µ = 1.40 mm^−^^1^
*c* = 25.3992 (11) Å	*T* = 293 K
β = 94.340 (1)°	Prism, colorless
*V* = 2168.57 (18) Å^3^	0.19 × 0.19 × 0.15 mm
*Z* = 4	

### Data collection

**Table:**

Rigaku R-AXIS RAPID IP diffractometer	4940 independent reflections
Radiation source: fine-focus sealed tube	3319 reflections with *I* > 2σ(*I*)
graphite	*R*_int_ = 0.040
ω scan	θ_max_ = 27.5°, θ_min_ = 3.2°
Absorption correction: multi-scan (*ABSCOR*; Higashi, 1995)	*h* = −15→15
*T*_min_ = 0.777, *T*_max_ = 0.817	*k* = −9→9
20669 measured reflections	*l* = −29→32

### Refinement

**Table:**

Refinement on *F*^2^	Primary atom site location: structure-invariant direct methods
Least-squares matrix: full	Secondary atom site location: difference Fourier map
*R*[*F*^2^ > 2σ(*F*^2^)] = 0.030	Hydrogen site location: inferred from neighbouring sites
*wR*(*F*^2^) = 0.082	H atoms treated by a mixture of independent and constrained refinement
*S* = 1.03	*w* = 1/[σ^2^(*F*_o_^2^) + (0.0405*P*)^2^ + 0.0996*P*] where *P* = (*F*_o_^2^ + 2*F*_c_^2^)/3
4940 reflections	(Δ/σ)_max_ = 0.001
355 parameters	Δρ_max_ = 0.33 e Å^−^^3^
18 restraints	Δρ_min_ = −0.40 e Å^−^^3^

### Special details

**Table:**

Geometry. All e.s.d.'s (except the e.s.d. in the dihedral angle between two l.s. planes) are estimated using the full covariance matrix. The cell e.s.d.'s are taken into account individually in the estimation of e.s.d.'s in distances, angles and torsion angles; correlations between e.s.d.'s in cell parameters are only used when they are defined by crystal symmetry. An approximate (isotropic) treatment of cell e.s.d.'s is used for estimating e.s.d.'s involving l.s. planes.

### Fractional atomic coordinates and isotropic or equivalent isotropic displacement parameters (Å^2^)

**Table:**

	*x*	*y*	*z*	*U*_iso_*/*U*_eq_	
Zn1	0.5000	0.5000	0.5000	0.02424 (10)	
Zn2	0.0000	0.5000	0.5000	0.02415 (10)	
S1	0.60479 (4)	0.93447 (8)	0.365706 (19)	0.02769 (13)	
S2	0.09496 (4)	0.94376 (8)	0.36609 (2)	0.02778 (13)	
O1	0.68413 (13)	0.8168 (2)	0.39683 (6)	0.0436 (4)	
O2	0.48651 (12)	0.8933 (2)	0.37348 (6)	0.0367 (4)	
O3	0.63007 (14)	1.1293 (2)	0.37462 (6)	0.0410 (4)	
O4	0.68090 (14)	0.8205 (2)	0.14198 (5)	0.0405 (4)	
O5	0.17678 (13)	0.8315 (2)	0.39749 (6)	0.0393 (4)	
O6	−0.02227 (12)	0.8971 (2)	0.37348 (6)	0.0385 (4)	
O7	0.11697 (13)	1.1408 (2)	0.37451 (5)	0.0359 (4)	
O8	0.17676 (14)	0.8359 (2)	0.14229 (6)	0.0412 (4)	
O1W	0.64682 (13)	0.6615 (2)	0.49333 (6)	0.0300 (3)	
O2W	0.43907 (16)	0.5631 (3)	0.42376 (6)	0.0425 (4)	
O3W	0.41407 (15)	0.7258 (2)	0.52682 (7)	0.0412 (4)	
O4W	0.15569 (14)	0.5586 (3)	0.47077 (8)	0.0463 (5)	
O5W	−0.08685 (14)	0.5987 (2)	0.43118 (6)	0.0361 (4)	
O6W	−0.01038 (14)	0.7610 (2)	0.53290 (6)	0.0349 (4)	
O7W	0.64137 (15)	0.9889 (2)	0.54413 (7)	0.0371 (4)	
O8W	0.16394 (15)	0.9973 (3)	0.52453 (8)	0.0410 (4)	
C1	0.62536 (16)	0.8899 (3)	0.29904 (7)	0.0243 (4)	
C2	0.73038 (17)	0.8248 (3)	0.28587 (8)	0.0290 (5)	
H2	0.7871	0.7971	0.3121	0.035*	
C3	0.74935 (17)	0.8018 (3)	0.23319 (8)	0.0296 (5)	
H3	0.8194	0.7591	0.2239	0.035*	
C4	0.66492 (17)	0.8420 (3)	0.19444 (7)	0.0271 (5)	
C5	0.55995 (17)	0.9073 (3)	0.20759 (8)	0.0323 (5)	
H5	0.5031	0.9340	0.1813	0.039*	
C6	0.54101 (17)	0.9321 (3)	0.26028 (8)	0.0299 (5)	
H6	0.4714	0.9771	0.2695	0.036*	
C7	0.11767 (16)	0.8998 (3)	0.29945 (8)	0.0254 (4)	
C8	0.21941 (18)	0.8220 (3)	0.28634 (8)	0.0315 (5)	
H8A	0.2734	0.7847	0.3128	0.038*	
C9	0.23990 (18)	0.8004 (3)	0.23389 (8)	0.0329 (5)	
H9	0.3080	0.7487	0.2250	0.039*	
C10	0.15988 (18)	0.8550 (3)	0.19468 (8)	0.0283 (5)	
C11	0.05795 (17)	0.9337 (3)	0.20744 (8)	0.0305 (5)	
H11A	0.0040	0.9707	0.1810	0.037*	
C12	0.03764 (17)	0.9561 (3)	0.26017 (8)	0.0283 (5)	
H12A	−0.0300	1.0092	0.2691	0.034*	
H4	0.7446 (14)	0.769 (4)	0.1384 (11)	0.068 (9)*	
H8	0.2355 (15)	0.769 (3)	0.1402 (11)	0.060 (9)*	
H11	0.659 (2)	0.694 (3)	0.4625 (5)	0.048 (8)*	
H12	0.642 (2)	0.762 (2)	0.5103 (9)	0.056 (8)*	
H21	0.459 (3)	0.657 (3)	0.4072 (11)	0.084 (11)*	
H22	0.3924 (17)	0.494 (3)	0.4062 (9)	0.048 (8)*	
H31	0.409 (2)	0.761 (4)	0.5581 (5)	0.054 (8)*	
H32	0.393 (2)	0.813 (3)	0.5066 (9)	0.065 (10)*	
H41	0.167 (2)	0.639 (3)	0.4480 (8)	0.058 (8)*	
H42	0.2171 (14)	0.511 (3)	0.4836 (10)	0.065 (10)*	
H51	−0.063 (2)	0.690 (3)	0.4144 (10)	0.073 (10)*	
H52	−0.122 (2)	0.518 (3)	0.4119 (9)	0.047 (8)*	
H61	0.0480 (18)	0.831 (4)	0.5340 (13)	0.102 (13)*	
H62	−0.037 (2)	0.768 (4)	0.5627 (6)	0.072 (10)*	
H71	0.7069 (12)	1.027 (4)	0.5550 (10)	0.053 (9)*	
H72	0.603 (2)	1.004 (4)	0.5705 (8)	0.084 (12)*	
H81	0.205 (2)	1.039 (4)	0.5508 (8)	0.059 (9)*	
H82	0.121 (3)	1.077 (4)	0.5089 (13)	0.112 (15)*	

### Atomic displacement parameters (Å^2^)

**Table:**

	*U*^11^	*U*^22^	*U*^33^	*U*^12^	*U*^13^	*U*^23^
Zn1	0.02731 (18)	0.0253 (2)	0.02019 (17)	−0.00203 (14)	0.00222 (14)	−0.00013 (14)
Zn2	0.02819 (18)	0.0218 (2)	0.02263 (18)	−0.00079 (14)	0.00322 (14)	0.00139 (14)
S1	0.0340 (3)	0.0312 (3)	0.0185 (3)	−0.0047 (2)	0.0060 (2)	0.0007 (2)
S2	0.0349 (3)	0.0284 (3)	0.0206 (3)	−0.0069 (2)	0.0056 (2)	0.0014 (2)
O1	0.0478 (9)	0.0573 (12)	0.0259 (8)	0.0082 (8)	0.0034 (7)	0.0106 (8)
O2	0.0347 (8)	0.0444 (10)	0.0323 (8)	−0.0053 (7)	0.0119 (7)	0.0013 (7)
O3	0.0598 (10)	0.0359 (10)	0.0293 (8)	−0.0147 (8)	0.0151 (8)	−0.0076 (7)
O4	0.0484 (10)	0.0537 (12)	0.0198 (7)	0.0188 (8)	0.0061 (7)	−0.0021 (7)
O5	0.0461 (9)	0.0444 (11)	0.0272 (8)	−0.0023 (8)	0.0004 (7)	0.0092 (7)
O6	0.0368 (8)	0.0438 (11)	0.0362 (9)	−0.0103 (7)	0.0122 (7)	0.0016 (8)
O7	0.0511 (9)	0.0290 (9)	0.0292 (8)	−0.0110 (7)	0.0131 (7)	−0.0067 (7)
O8	0.0520 (11)	0.0486 (11)	0.0235 (8)	0.0167 (9)	0.0068 (8)	−0.0010 (7)
O1W	0.0377 (8)	0.0267 (9)	0.0262 (8)	−0.0057 (7)	0.0065 (7)	0.0017 (7)
O2W	0.0637 (12)	0.0380 (11)	0.0237 (9)	−0.0182 (9)	−0.0102 (8)	0.0058 (8)
O3W	0.0605 (11)	0.0352 (11)	0.0288 (9)	0.0136 (9)	0.0087 (8)	−0.0025 (8)
O4W	0.0312 (9)	0.0519 (12)	0.0574 (12)	0.0040 (9)	0.0129 (9)	0.0285 (10)
O5W	0.0480 (10)	0.0315 (10)	0.0274 (9)	−0.0078 (8)	−0.0060 (8)	0.0057 (8)
O6W	0.0509 (10)	0.0255 (9)	0.0297 (9)	−0.0042 (8)	0.0112 (8)	−0.0049 (7)
O7W	0.0358 (9)	0.0393 (10)	0.0370 (9)	−0.0047 (8)	0.0078 (8)	−0.0056 (8)
O8W	0.0379 (10)	0.0417 (11)	0.0431 (11)	−0.0049 (8)	0.0017 (9)	−0.0040 (9)
C1	0.0297 (11)	0.0248 (12)	0.0189 (9)	−0.0033 (9)	0.0048 (9)	0.0011 (8)
C2	0.0312 (11)	0.0305 (13)	0.0247 (10)	0.0054 (9)	−0.0023 (9)	−0.0004 (9)
C3	0.0303 (11)	0.0314 (13)	0.0274 (11)	0.0086 (9)	0.0053 (9)	−0.0025 (9)
C4	0.0372 (12)	0.0241 (12)	0.0202 (10)	0.0030 (9)	0.0037 (9)	−0.0026 (9)
C5	0.0308 (11)	0.0413 (15)	0.0241 (11)	0.0051 (10)	−0.0015 (9)	−0.0007 (10)
C6	0.0260 (10)	0.0362 (13)	0.0278 (11)	0.0016 (10)	0.0045 (9)	−0.0003 (9)
C7	0.0308 (11)	0.0221 (12)	0.0235 (10)	−0.0046 (9)	0.0024 (9)	−0.0016 (9)
C8	0.0347 (12)	0.0315 (13)	0.0277 (11)	0.0052 (10)	−0.0019 (10)	0.0004 (9)
C9	0.0343 (12)	0.0332 (13)	0.0316 (12)	0.0089 (10)	0.0050 (10)	−0.0027 (10)
C10	0.0381 (12)	0.0251 (12)	0.0221 (10)	0.0008 (9)	0.0057 (9)	−0.0004 (9)
C11	0.0308 (11)	0.0332 (13)	0.0267 (11)	0.0052 (10)	−0.0036 (9)	0.0004 (9)
C12	0.0273 (11)	0.0285 (12)	0.0296 (12)	0.0000 (9)	0.0046 (9)	−0.0021 (9)

### Geometric parameters (Å, °)

**Table:**

Zn1—O2W	2.0654 (16)	O4W—H41	0.840 (10)
Zn1—O2W^i^	2.0654 (16)	O4W—H42	0.847 (10)
Zn1—O3W	2.0692 (16)	O5W—H51	0.846 (10)
Zn1—O3W^i^	2.0692 (16)	O5W—H52	0.849 (10)
Zn1—O1W^i^	2.1087 (15)	O6W—H61	0.854 (10)
Zn1—O1W	2.1087 (15)	O6W—H62	0.842 (10)
Zn2—O4W^ii^	2.0763 (15)	O7W—H71	0.847 (10)
Zn2—O4W	2.0763 (15)	O7W—H72	0.846 (10)
Zn2—O6W	2.0780 (16)	O8W—H81	0.849 (10)
Zn2—O6W^ii^	2.0780 (16)	O8W—H82	0.848 (10)
Zn2—O5W	2.0849 (16)	C1—C6	1.380 (3)
Zn2—O5W^ii^	2.0849 (16)	C1—C2	1.390 (3)
S1—O2	1.4549 (14)	C2—C3	1.383 (3)
S1—O1	1.4555 (17)	C2—H2	0.9300
S1—O3	1.4598 (17)	C3—C4	1.377 (3)
S1—C1	1.7587 (19)	C3—H3	0.9300
S2—O6	1.4495 (15)	C4—C5	1.390 (3)
S2—O5	1.4542 (16)	C5—C6	1.385 (3)
S2—O7	1.4666 (16)	C5—H5	0.9300
S2—C7	1.763 (2)	C6—H6	0.9300
O4—C4	1.369 (2)	C7—C12	1.382 (3)
O4—H4	0.851 (10)	C7—C8	1.389 (3)
O8—C10	1.367 (2)	C8—C9	1.381 (3)
O8—H8	0.849 (10)	C8—H8A	0.9300
O1W—H11	0.843 (10)	C9—C10	1.378 (3)
O1W—H12	0.849 (10)	C9—H9	0.9300
O2W—H21	0.845 (10)	C10—C11	1.391 (3)
O2W—H22	0.846 (10)	C11—C12	1.388 (3)
O3W—H31	0.840 (10)	C11—H11A	0.9300
O3W—H32	0.841 (10)	C12—H12A	0.9300
			
O2W—Zn1—O2W^i^	180.0	Zn1—O3W—H31	128.5 (19)
O2W—Zn1—O3W	89.34 (7)	Zn1—O3W—H32	121.7 (19)
O2W^i^—Zn1—O3W	90.66 (7)	H31—O3W—H32	108 (3)
O2W—Zn1—O3W^i^	90.66 (7)	Zn2—O4W—H41	125.1 (18)
O2W^i^—Zn1—O3W^i^	89.34 (7)	Zn2—O4W—H42	122.1 (18)
O3W—Zn1—O3W^i^	180.0	H41—O4W—H42	112 (2)
O2W—Zn1—O1W^i^	88.23 (6)	Zn2—O5W—H51	122 (2)
O2W^i^—Zn1—O1W^i^	91.77 (6)	Zn2—O5W—H52	115.8 (18)
O3W—Zn1—O1W^i^	89.32 (7)	H51—O5W—H52	114 (3)
O3W^i^—Zn1—O1W^i^	90.68 (7)	Zn2—O6W—H61	119 (2)
O2W—Zn1—O1W	91.77 (6)	Zn2—O6W—H62	117 (2)
O2W^i^—Zn1—O1W	88.23 (6)	H61—O6W—H62	107 (3)
O3W—Zn1—O1W	90.68 (7)	H71—O7W—H72	104 (3)
O3W^i^—Zn1—O1W	89.32 (7)	H81—O8W—H82	114 (3)
O1W^i^—Zn1—O1W	180.0	C6—C1—C2	120.75 (18)
O4W^ii^—Zn2—O4W	180.0	C6—C1—S1	120.10 (15)
O4W^ii^—Zn2—O6W	87.85 (7)	C2—C1—S1	118.98 (16)
O4W—Zn2—O6W	92.15 (7)	C3—C2—C1	119.08 (19)
O4W^ii^—Zn2—O6W^ii^	92.15 (7)	C3—C2—H2	120.5
O4W—Zn2—O6W^ii^	87.85 (7)	C1—C2—H2	120.5
O6W—Zn2—O6W^ii^	180.0	C4—C3—C2	120.31 (18)
O4W^ii^—Zn2—O5W	88.76 (7)	C4—C3—H3	119.8
O4W—Zn2—O5W	91.24 (7)	C2—C3—H3	119.8
O6W—Zn2—O5W	89.07 (7)	O4—C4—C3	121.75 (18)
O6W^ii^—Zn2—O5W	90.93 (7)	O4—C4—C5	117.61 (19)
O4W^ii^—Zn2—O5W^ii^	91.24 (7)	C3—C4—C5	120.64 (18)
O4W—Zn2—O5W^ii^	88.76 (7)	C6—C5—C4	119.2 (2)
O6W—Zn2—O5W^ii^	90.93 (7)	C6—C5—H5	120.4
O6W^ii^—Zn2—O5W^ii^	89.07 (7)	C4—C5—H5	120.4
O5W—Zn2—O5W^ii^	180.0	C1—C6—C5	120.01 (19)
O2—S1—O1	112.93 (10)	C1—C6—H6	120.0
O2—S1—O3	111.38 (10)	C5—C6—H6	120.0
O1—S1—O3	111.66 (10)	C12—C7—C8	120.14 (19)
O2—S1—C1	107.25 (9)	C12—C7—S2	119.64 (15)
O1—S1—C1	106.56 (9)	C8—C7—S2	120.03 (16)
O3—S1—C1	106.63 (9)	C9—C8—C7	119.7 (2)
O6—S2—O5	113.46 (9)	C9—C8—H8A	120.2
O6—S2—O7	111.65 (9)	C7—C8—H8A	120.2
O5—S2—O7	111.33 (10)	C10—C9—C8	120.29 (19)
O6—S2—C7	107.33 (9)	C10—C9—H9	119.9
O5—S2—C7	106.41 (9)	C8—C9—H9	119.9
O7—S2—C7	106.16 (9)	O8—C10—C9	122.13 (18)
C4—O4—H4	109.9 (19)	O8—C10—C11	117.43 (19)
C10—O8—H8	107.3 (19)	C9—C10—C11	120.44 (18)
Zn1—O1W—H11	115.8 (17)	C10—C11—C12	119.2 (2)
Zn1—O1W—H12	110.2 (17)	C10—C11—H11A	120.4
H11—O1W—H12	105 (2)	C12—C11—H11A	120.4
Zn1—O2W—H21	124 (2)	C7—C12—C11	120.23 (19)
Zn1—O2W—H22	121.9 (19)	C7—C12—H12A	119.9
H21—O2W—H22	114 (3)	C11—C12—H12A	119.9
			
O2—S1—C1—C6	37.7 (2)	O6—S2—C7—C12	44.6 (2)
O1—S1—C1—C6	158.94 (18)	O5—S2—C7—C12	166.37 (17)
O3—S1—C1—C6	−81.67 (19)	O7—S2—C7—C12	−74.94 (19)
O2—S1—C1—C2	−146.98 (17)	O6—S2—C7—C8	−140.39 (17)
O1—S1—C1—C2	−25.8 (2)	O5—S2—C7—C8	−18.6 (2)
O3—S1—C1—C2	93.60 (18)	O7—S2—C7—C8	100.09 (18)
C6—C1—C2—C3	−0.3 (3)	C12—C7—C8—C9	−0.3 (3)
S1—C1—C2—C3	−175.53 (16)	S2—C7—C8—C9	−175.31 (17)
C1—C2—C3—C4	−0.4 (3)	C7—C8—C9—C10	−0.2 (3)
C2—C3—C4—O4	−179.9 (2)	C8—C9—C10—O8	−179.9 (2)
C2—C3—C4—C5	0.5 (3)	C8—C9—C10—C11	0.4 (3)
O4—C4—C5—C6	−179.5 (2)	O8—C10—C11—C12	−179.8 (2)
C3—C4—C5—C6	0.1 (3)	C9—C10—C11—C12	−0.1 (3)
C2—C1—C6—C5	0.9 (3)	C8—C7—C12—C11	0.6 (3)
S1—C1—C6—C5	176.08 (17)	S2—C7—C12—C11	175.62 (17)
C4—C5—C6—C1	−0.8 (3)	C10—C11—C12—C7	−0.4 (3)

Symmetry codes: (i) −*x*+1, −*y*+1, −*z*+1; (ii) −*x*, −*y*+1, −*z*+1.

### Hydrogen-bond geometry (Å, °)

**Table:**

*D*—H···*A*	*D*—H	H···*A*	*D*···*A*	*D*—H···*A*
O1w—H11···O1	0.84 (1)	1.93 (1)	2.763 (2)	169 (3)
O1w—H12···O7w	0.85 (1)	1.86 (1)	2.707 (2)	175 (2)
O2w—H21···O2	0.85 (1)	1.95 (1)	2.792 (2)	172 (3)
O2w—H22···O4^iii^	0.85 (1)	1.92 (1)	2.745 (2)	166 (3)
O3w—H31···O3^iv^	0.84 (1)	1.97 (1)	2.800 (2)	168 (3)
O3w—H32···O7w^iv^	0.84 (1)	1.95 (1)	2.790 (2)	174 (3)
O4w—H41···O5	0.84 (1)	1.91 (1)	2.742 (2)	174 (3)
O4w—H42···O1w^i^	0.85 (1)	2.08 (1)	2.914 (2)	167 (3)
O5w—H51···O6	0.85 (1)	1.91 (1)	2.755 (2)	175 (3)
O5w—H52···O8^v^	0.85 (1)	1.98 (1)	2.819 (2)	168 (3)
O6w—H61···O8w	0.85 (1)	1.85 (1)	2.698 (2)	170 (3)
O6w—H62···O7^vi^	0.84 (1)	2.03 (1)	2.8401 (19)	163 (3)
O4—H4···O7^iii^	0.85 (1)	1.93 (1)	2.777 (2)	175 (3)
O8—H8···O3^iii^	0.85 (1)	1.95 (1)	2.788 (2)	172 (3)

Symmetry codes: (iii) −*x*+1, *y*−1/2, −*z*+1/2; (iv) −*x*+1, −*y*+2, −*z*+1; (i) −*x*+1, −*y*+1, −*z*+1; (v) −*x*, *y*−1/2, −*z*+1/2; (vi) −*x*, −*y*+2, −*z*+1.

## References

[bb1] Barbour, L. J. (2001). *J. Supramol. Chem.* **1**, 189–191.

[bb2] Du, J.-M., Li, Q., Li, W., Lin, H.-M. & Guo, G.-C. (2007). *Acta Cryst.* E**63**, m2597.

[bb3] Higashi, T. (1995). *ABSCOR*. Rigaku Corporation, Tokyo, Japan.

[bb4] Kosnic, J., McClymont, E. L., Hodder, R. A. & Squattrito, P. J. (1992). *Inorg. Chim. Acta*, **201**, 143–151.

[bb5] Rigaku (1998). *RAPID-AUTO*. Rigaku Corporation, Tokyo, Japan.

[bb6] Rigaku/MSC (2002). *CrystalClear*. Rigaku/MSC Inc., The Woodlands, Texas, USA.

[bb7] Sheldrick, G. M. (2008). *Acta Cryst.* A**64**, 112–122. 10.1107/S0108767307043930 18156677

[bb8] Westrip, S. P. (2009). *publCIF*. In preparation.

